# Detection of *Staphylococcus* and *Streptococcus* Resistant to Antibiotics in Subclinical Bovine Mastitis in Ecuador

**DOI:** 10.3390/vetsci13060579

**Published:** 2026-06-13

**Authors:** Andrea Flores-Garzón, Kevin Guevara, Andrea Carrera-González, Nina Espinosa de los Monteros-Silva, Carolina Proaño-Bolaños, Pedro Barba

**Affiliations:** 1 Carrera de Biotecnología, Facultad de Ingeniería en Ciencias Agropecuarias y Ambientales, Universidad Técnica del Norte, Ibarra 100105, Ecuador; anfloresg@utn.edu.ec (A.F.-G.); keguevarai@utn.edu.ec (K.G.); 2 Laboratorio de Biología Molecular y Bioquímica, Universidad Regional Amazónica Ikiam, Tena 150102, Ecuador; andrea.carrera@ikiam.edu.ec (A.C.-G.); nina.espinosadelosmonteros@ikiam.edu.ec (N.E.d.l.M.-S.); 3 Biomolecules Discovery Group, Laboratorio de Biología Molecular y Bioquímica, Universidad Regional Amazónica Ikiam, Tena 150102, Ecuador; carolina.proano@ikiam.edu.ec

**Keywords:** subclinical bovine mastitis, *Staphylococcus*, *Streptococcus*, antimicrobial resistance, dairy industry, Ecuador

## Abstract

Subclinical bovine mastitis (SBM) is an inflammatory condition of the udder that remains a major concern for the dairy industry due to its high incidence and the direct and indirect associated costs. Antibiotics are widely used for prophylaxis and therapy in livestock, especially for SBM. However, overuse and misuse have contributed to the emergence of antimicrobial resistance (AMR), enabling resistant bacteria to enter the food chain and potentially spread to humans. This study aimed to detect antibiotic-resistant *Staphylococcus* and *Streptococcus* associated with SBM in dairy cows from Pioter, north-central Ecuador. For this, a commercial screening test, morphological and biochemical assays, standard culture techniques, mass spectrometry, and PCR (polymerase chain reaction) were applied. Among 99 isolates, 77 were *Staphylococcus* and 22 were *Streptococcus*. Among the identified *Staphylococcus* isolates, *S. aureus* was the predominant species (36.4%). Resistance in *Staphylococcus* exceeded 70% for fosfomycin and was under 30% for the other antibiotics tested. In *Streptococcus*, *S. uberis* predominated (54.5%), with resistance primarily to penicillin and tetracycline (>50%). PCR identified *mecA*, *nuc*, and *lukSF-PV* genes in 7.8%, 29.9%, and 6.5% of *Staphylococcus* isolates, respectively. In *Streptococcus*, the *ermB* and *blaZ* genes were found in 18.2% and 50% of isolates, respectively. These data provide a baseline on SBM-associated AMR in the study area and highlight the need for ongoing surveillance and improved milking practices to mitigate risks to the dairy sector and public health.

## Simple Summary

Subclinical bovine mastitis (SBM) represents a major challenge for the dairy industry with a global impact. This infection represents substantial economic losses due to lower milk quality, milk discard, animal isolation, or death. Ecuador produced an estimated 5.31 million liters of milk in 2024, with the Andean highlands contributing most of the production. It has been demonstrated that routine and local antimicrobial resistance (AMR) surveillance, integrating phenotypic testing with molecular assays, could improve the management of this condition. Here, a cross-sectional study was conducted in six farms in the northern part of Ecuador, where milk samples from 252 cattle units were analyzed. Using a standard testing technique designed to detect SBM, an important prevalence of this infection was found. Several species of *Staphylococcus* and *Streptococcus* genera were detected using mass spectrometry, with *S. aureus*, *S. warneri*, *S. epidermidis*, and *S. uberis* being the most prevalent. Among *Staphylococcus* isolates, important resistance levels to fosfomycin and oxacillin/cefoxitin were observed, while for *Streptococcus* isolates, significant resistance levels to tetracycline, gentamicin, and penicillin were also observed. The phenotypic resistance found here correlated with the presence of genetic resistance elements, highlighting the need for deeper molecular analyses to detect additional resistance determinants. The findings underscore the importance of continuous surveillance, as well as the required implementation of improved milking hygiene practices to reduce the infection pressure, antibiotic use, and associated One Health risk.

## 1. Introduction

Subclinical bovine mastitis (SBM) is an inflammatory disease of the mammary gland in dairy cows [1]. Three main factors contribute to the development of SBM: udder colonization by microorganisms, poor nutrition, and suboptimal herd management [2]. During lactation, nutritional demands increase to sustain milk synthesis; if intake does not meet requirements, cows can develop cellular and humoral immunosuppression, increasing their susceptibility to intramammary infection [3]. Management factors at the herd level, including high stocking density, inadequate ventilation, and hot and humid environments, further promote the growth and transmission of mastitis pathogens [4]. SBM has substantial economic impact due to direct (treatment, discarded milk, animal isolation, or death) and indirect (reduced yield, altered composition, and lower milk quality) losses [5,6].

Bacterial intramammary infections underlying SBM are commonly classified as contagious or environmental microorganisms [1]. Predominant contagious pathogens include *Staphylococcus aureus*, *Streptococcus agalactiae*, and *Corynebacterium bovis*, whereas *Escherichia coli*, *Klebsiella* spp., *Streptococcus uberis*, and *Streptococcus dysgalactiae* are major environmental bacteria [5]. Antibiotics remain the mainstay for the prophylaxis and therapy of SBM in the dairy sector [1]. However, AMR among mastitis pathogens is a growing concern, driven by overuse and misuse in livestock [2,7]. Resistant bacteria can enter the food chain, posing a risk of dissemination to humans. From a One Health perspective, mastitis pathogens can cross species barriers via contaminated milk or meat, creating a public health threat [2].

In farms, screening of mastitis typically relies on the California Mastitis Test (CMT) and somatic cell counts, whereas culture-based identification and phenotypic susceptibility testing are needed for targeted control, ideally supported by molecular techniques for species confirmation and resistance determinant detection [2].

Ecuador produced an estimated 5.31 million liters of milk in 2024, ~74.85% for fluid sale and the remainder for calf feeding or processing at the farm level. The Andean highlands contribute the most, accounting for 77.7% of the national output, followed by the Pacific coast with 18.3% and the Amazon basin and others with 4% [8,9,10]. Historical data indicate a high risk of SBM in Ecuadorian herds, which is linked to deficiencies in milking hygiene, equipment maintenance, disinfectant handling, etiologic misdiagnosis, and antibiotic stewardship in farms [11].

In response to the urgent need to curb antimicrobial misuse and enhance surveillance of zoonotic antimicrobial resistance (AMR), this study aimed to identify the predominant *Staphylococcus* and *Streptococcus* species associated with subclinical bovine mastitis (SBM) in dairy cattle from farms in Pioter (Carchi, northern Ecuador), as well as characterize their antimicrobial susceptibility profiles and investigated resistance genetic determinants to establish a local baseline to support future intervention strategies.

## 2. Materials and Methods

### 2.1. Location of Sampling, Study Design, and Sample Size

Sampling was conducted between November 2019 and March 2020 in the Pioter parish (Carchi Province, Ecuador; 0°38′43″ N, 77°47′02″ W; ~3000 m.a.s.l.), with a mean annual temperature of 16 °C and mean annual rainfall of 1721 mm. In 2020, 4471 cattle units were registered in Pioter [12]. A sample size of 252 cattle units was calculated using a standard statistical formula, and the units were randomly selected from six dairy farms. Milk samples were collected during routine milking procedures, avoiding direct contact with the herd.

### 2.2. Detection of SBM Using California Mastitis Test (CMT)

The CMT was used as a preliminary screen for SBM [13]. From each cow, 2 mL of foremilk from each quarter was mixed with 2 mL of CMT reagent (3% sodium lauryl sulfate with 1:10,000 bromocresol purple, pH 7.0–7.5). The results were interpreted according to the degree of viscosity increase and gelation (Table 1) [6,14,15,16].

Individuals presenting at least one positive quarter were classified as SBM-positive. From each CMT-positive cow, 15 mL of milk was pooled across quarters, which was collected aseptically before routine milking processes; stored at 4 °C; and processed immediately at Laboratorio de Biotecnología Aplicada of Universidad Técnica del Norte, Ibarra, Ecuador.

### 2.3. Isolation of Staphylococcus *spp.* and Streptococcus *spp.*

From each milk sample, 100 µL was inoculated onto Mannitol Salt Agar and 5% sheep Blood Agar to isolate *Staphylococcus* spp. and *Streptococcus* spp., respectively [17]. Plates were incubated at 37 °C for 24–48 h [18,19,20]. One to two colonies with typical *Staphylococcus* and *Streptococcus* morphologies were subcultured on Baird–Parker Agar and Blood Agar, respectively, to obtain axenic cultures.

### 2.4. Morphological and Biochemical Characterization of Isolates

Gram staining, catalase, and coagulase tests were performed. Hemolytic activity was assessed for *Staphylococcus* spp. discrimination [21]. For the catalase test, a drop of 3% H_2_O_2_ was applied to 1–2 colonies [22]. For the coagulase test, 1 mL of blood plasma was incubated with 1–2 colonies at 37 °C for 24 h [23].

### 2.5. Species Confirmation Using Mass Spectrometry

Isolates with morphology and biochemical characteristics consistent with *Staphylococcus* or *Streptococcus* were identified using Matrix-Assisted Laser Desorption Ionization Time-of-Flight (MALDI-TOF MS) (Axima Confidence, Shimadzu, Kyoto, Japan). The assays were performed at the Laboratorio de Biología Molecular y Bioquímica of Universidad Regional Amazónica Ikiam, Tena, Ecuador, using α-cyano-4-hydroxycinnamic acid as the matrix in positive linear mode. MALDI-MS Application (v2.9.4.1) and SARAMIS (v4.1.0.9) were used for spectral processing and microorganism identification. Three technical replicates per isolate were assessed.

### 2.6. Antibiotic Susceptibility Profile of Microorganisms

The Kirby–Bauer disk diffusion test was performed following standardized methods [24,25,26]. Cultures on Mueller–Hinton Agar (MHA) or MHA + 5% sheep blood were incubated at 37 °C for 24–48 h. For *Staphylococcus* isolates, the following agents were tested: cefoxitin (FOX), oxacillin (OXA), trimethoprim–sulfamethoxazole (SXT), erythromycin (ERY), clindamycin (CLI), rifampicin (RIF), gentamicin (GEN), ciprofloxacin (CIP), tetracycline (TET), fosfomycin (FOS), and linezolid (LNZ). For *Streptococcus* isolates: penicillin (PEN), ceftriaxone (CRO), SXT, ERY, CLI, RIF, GEN, CIP, TET, and LNZ. Zone diameters were recorded and interpreted accordingly (Table 2) [25,26].

### 2.7. Molecular Detection of Antibiotic Resistance and Virulence Genes

Resistance (*mecA*, *tetL*, *ermB*, *blaZ*) and virulence (*nuc*, *lukSF-PV*) genes were detected using PCR (Table 3). Genomic DNA was extracted with the Wizard^®^ Genomic DNA Purification Kit Promega Corporation, Madison, WI, USA [27], according to manufacturer specifications. The DNA quality was evaluated using spectrophotometry and 1% agarose gel electrophoresis.

PCR fragments were amplified in 25 µL reactions with 12.5 µL GoTaq^®^ Green Master Mix (Madison, WI, USA), with a final concentration of 3 mM MgCl_2_, 0.2 µM of each primer (Table 3), and 20 ng/µL of DNA [34]. The thermocycling program was 94 °C, 5 min; 35 cycles of 94 °C, 1 min; gene-specific annealing (Table 2), 30 s; 72 °C, 30 s; and a final extension 72 °C, 5 min. Amplicons were visualized on 1% agarose gel electrophoresis. 

### 2.8. Statistical Analysis

Data were initially analyzed using descriptive statistics (frequencies and percentages). Subsequently, Fisher’s exact tests and Kendall’s correlation coefficients were used to assess the association between phenotypic and genotypic resistance profiles. A significance level of *p* < 0.05 was applied to all analyses. The software M365 Copilot (GPT‑5 chat, OpenAI) was used as an AI-assisted tools to support language and grammar editing of the manuscript. All scientific content, interpretations, and conclusions remain the responsibility of the authors.

## 3. Results and Discussion

### 3.1. Subclinical Bovine Mastitis Evaluation

Eighty cattle presented a positive CMT in at least one quarter, representing a 31.7% SBM prevalence (Table 4).

These data are similar to reports from Pichincha, Carchi, and Manabí in Ecuador, where 35–38% of infected individuals were reported [35,36,37,38] and are consistent with data from neighboring Colombia [39]; a higher prevalence of infected cows (72.25%) was observed in Tamburco (Perú) [40]. In Ecuador, the highest prevalences were found in Cuenca (42.1%) [41], Cayambe (45.5%) [42], Paquiestancia (64%), and El Pangui (65.4%) [43,44]. Multiple factors influence the development of SBM, including cow age, size of the agricultural production unit, farm altitude, milking method (manual vs. mechanical), milking frequency, grazing systems, and lactation stage. However, sanitation throughout the milking routine is particularly critical [40,41,45,46]. Prior studies show that there is no single cause of SBM. For example, some reports specify that over 50% of SBM cases are associated with mechanical milking, cattle over five years of age, and poor milking practices [40,41,46]. In our study, farms 4, 5, and 6 had prevalences lower than 30% SBM despite sharing a similar milking method with the other farms (Table 4). While these results cannot be attributed to the milking technique alone, farms 4, 5, and 6 consistently applied mandatory post-milking udder disinfection, with farms 4 and 6 also using disposable paper for drying, avoiding cross-contamination associated with reusable cloths when not properly disinfected [47,48,49,50,51,52]. This good milking practice was applied less frequently on farms 1, 2, and 3.

### 3.2. Staphylococcus *spp.* and Streptococcus *spp.* Identification

**Figure 1:**
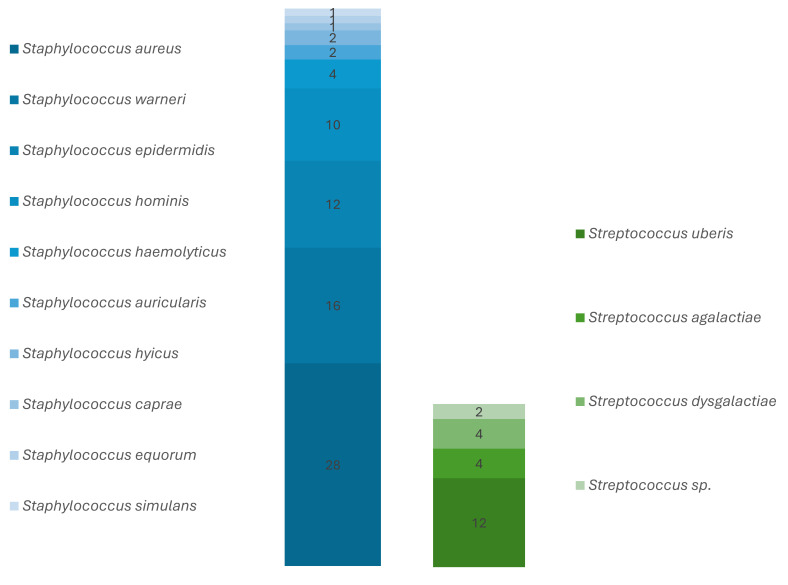
MALDI-TOF MS species identification. Number of isolates identified in each species.

Using standard phenotypic methods, we characterized 99 isolates as *Staphylococcus* or *Streptococcus*. The catalase test identified 77 isolates as *Staphylococcus*, of which 49 (63.6%) were coagulase-negative (CoNS). The remaining 22 catalase-negative isolates were assigned as *Streptococcus*. Species identification using MALDI-TOF MS correctly resolved 97.9% of strains (Figure 1).

Within *Staphylococcus*, *S. aureus* was the predominant species, followed by *S. warneri* and *S. epidermidis*. These findings agree with the recognized role of *S. aureus* as a major mastitis pathogen worldwide [4,53,54,55]. *S. epidermidis* is a common component of human skin microbiota, easily increasing the risk of cow udder infection transmission during the milking process and causing teat meatus and cistern colonization [56,57,58]. Moreover, this microorganism can proliferate in udder wounds, particularly in teat lesions caused by mechanical milking [59,60]. Consequently, rigorous disinfection protocols for equipment and hands are essential to limit cross-contamination [61]. The remaining *Staphylococcus* species (CoNS) (Figure 1) are often termed minor pathogens; however, their detection is relevant since they can behave as opportunists [62,63,64,65].

Among *Streptococcus*, *S. uberis* was the predominant species found here (Figure 1). As a ubiquitous microorganism, *S. uberis* is the most frequent cause of SBM globally among this genus and is responsible for over one-third of all cases [62,66,67,68]. It is influenced by hygiene and environmental conditions, and by its ability to persist in bedding and resting areas [69,70]. *S. uberis* can colonize the gastrointestinal tract, survive after fecal shedding, and infect mammary glands during lactation and the dry period, sometimes remaining asymptomatic [71,72,73]. This emphasizes the need for continuous monitoring throughout all phases in the dairy industry.

### 3.3. Antibiotic Susceptibility Profile of Isolates

**Figure 2:**
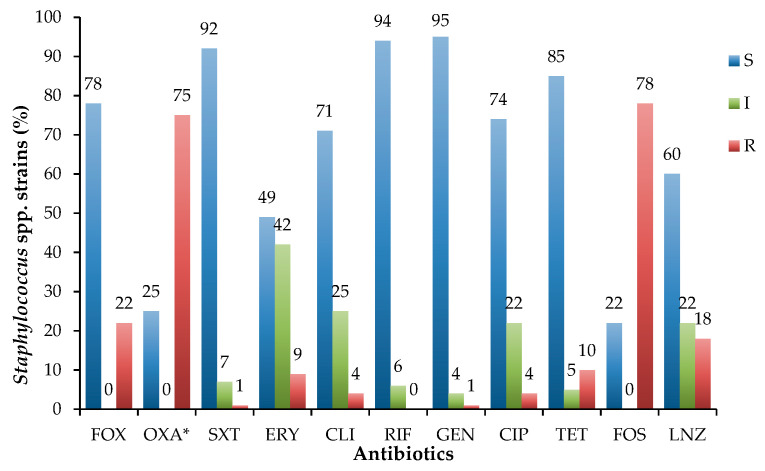
Antibiotic susceptibility profile of *Staphylococcus* spp. isolates. FOX: cefoxitin; OXA: oxacillin; SXT: sulfamethoxazole; ERY: erythromycin; CLI: clindamycin; RIF: rifampicin; GEN: gentamicin; CIP: ciprofloxacin; TET: tetracycline; FOS: fosfomycin; LNZ: linezolid; S: susceptible; I: intermediate; R: resistant. n = 77. * Oxacillin was tested only in *Staphylococcus epidermidis* isolates (n = 12).

Antibiotic-resistant *Staphylococcus* strains have been described in clinical, community, and environmental settings as an important health risk factor. Furthermore, antibiotic-resistant *Staphylococcus* livestock-associated strains have been reported in many regions to cause SBM [74,75,76,77]. In this study, 78% of the *Staphylococcus* isolates were resistant to fosfomycin (FOS) (Figure 2). Despite the resistance level to the remaining antibiotics being under 30%, the combination of high potential transmissibility and invasiveness can drive rapid dissemination throughout a herd, amplifying the burden to dairy cows [16,76].

The highest resistance level in *Staphylococcus* was against FOS, followed by FOX and LZD (Figure 2). *Staphylococcus* has shown FOS resistance primarily by carrying the *fosA* resistance gene or by cellular uptake reduction through mutations in the hexose phosphate (UhpT) and glycerol-3-phosphate (GlpT) systems [78,79,80]. While global reports of FOS resistance have historically been less frequent, the dependence on these drugs to combat multidrug-resistant pathogens has raised concerns, including SBM [81]. The use of FOS has risen year over year, heightening the risk of selecting FOS resistance [82,83,84,85]. Resistance to FOS has been described as more prevalent in healthcare-associated MRSA (70.7%) compared with community-onset MRSA (30.2%) [86]; therefore, a human health source could be involved here. Our results further reinforce the need to reduce antibiotic exposure at the farm level and the need for deeper molecular analysis. For OXA, the results here are similar to other reports, which indicate 47–84% resistant strains [1,87]. Furthermore, FOX is a β-lactam antibiotic, which is one of the most common drug types used against bovine infections [88,89,90,91,92,93,94]. A study in China reported 30% FOX resistance [95]. There are no exhaustive studies for FOS or FOX resistance in SBM-causing *Staphylococcus* in Ecuador; therefore, the present work can be used as a base for future studies that focus on antibiotic resistance in *Staphylococcus* infection.

**Figure 3:**
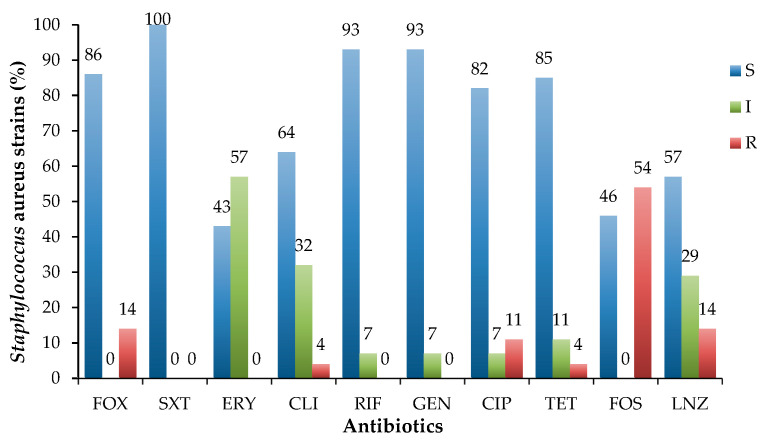
Antibiotic susceptibility profile of *Staphylococcus aureus* isolates. FOX: cefoxitin; SXT: sulfamethoxazole; ERY: erythromycin; CLI: clindamycin; RIF: rifampicin; GEN: gentamicin; CIP: ciprofloxacin; TET: tetracycline; FOS: fosfomycin; LNZ: linezolid; S: susceptible; I: intermediate; R: resistant. n = 28.

No resistance was displayed against RIF (Figure 2). Studies from Venezuela, Spain, and Romania reported 100% of *S. aureus* and *S. saprophyticus* strains being susceptible to RIF [14,96,97]. Rifampicin has been considered a last-resort drug to treat *S. aureus* infections, especially MRSA strains [98]. RIF penetrates cells, tissues, and biofilms more effectively than β-lactams and glycopeptides [99,100]; consequently, effective synergy with other drugs has been reported regarding the treatment of invasive and persistent infections [98]. Furthermore, in human assays, RIF has demonstrated good action in treating mastitis [101,102]. In this study, *S. aureus* showed resistance against six of the eleven antibiotics tested. Up to 50% of isolates were cataloged as FOS-resistant microorganisms, followed by resistance to FOX, LZD, CIP, TET, and CLI (Figure 3).

Across the other *Staphylococcus* species identified (Figure 1), resistance was generally low, except to FOS and FOX, with elevated resistance levels. The high levels of FOS-resistant CoNS isolates observed here alert us to a potential environmental reservoir of resistance determinants, demanding further studies given its potential health risk [103,104]. Overall, the prevalence of antibiotic-resistant *Staphylococcus* in our study was lower than values reported in other settings. For example, prior reports describe 25% SXT resistance in the USA [105]; 85% OXA, 34% TET, and 33% CIP resistance in China [106]; 11.6% and 33.8% ERY resistance in Argentina and Brazil, respectively [107,108]; 76.7% TET resistance in Iran [109]; and 25% FOX resistance in Kenya [110]. However, our values were still higher than other reports, such as 5.7% ERY resistance in Uruguay and 3.6% OXA resistance in Colombia [111,112].

Therefore, even though resistance to some agents was low, particular attention should be paid to linezolid (LZD), the first oxazolidinone approved for clinical use [113]. LZD is effective against the principal Gram-positive pathogens and remains active against many multidrug-resistant organisms in this group [114]. Nevertheless, a 2012 analysis of data collected from 2002 to 2010 documented a rising incidence of LZD resistance over time [115]. These observations call for continued surveillance of the prevalence of LZD-resistant isolates. In addition, the antibiotics showing ≤ 10% resistance in this study (Figure 2 and Figure 3) are broad-spectrum antibiotics widely used in veterinary and human medicine, drawing our attention to future analysis [116,117]. Finally, isolates categorized as intermediate deserve close attention because inadequate or suboptimal dosing may facilitate their progression to full resistance [118,119,120].

**Figure 4:**
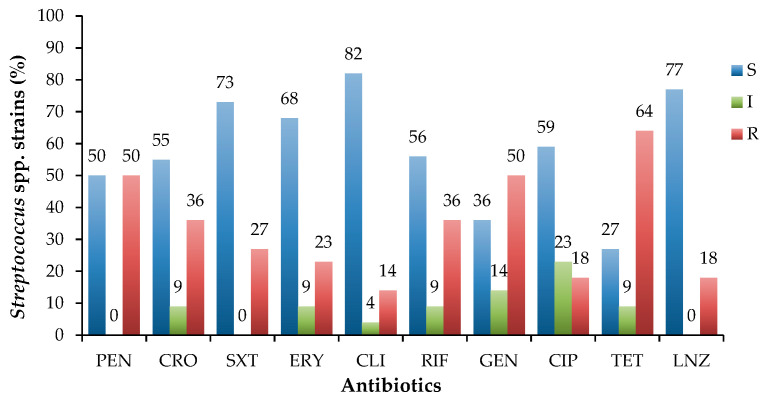
Antibiotic susceptibility profile of *Streptococcus* spp. isolates. PEN: penicillin; CRO: ceftriaxone; SXT: sulfamethoxazole; ERY: erythromycin; CLI: clindamycin; RIF: rifampicin; GEN: gentamicin; CIP: ciprofloxacin; TET: tetracycline; LNZ: linezolid; S: susceptible; I: intermediate; R: resistant. n = 22.

In *Streptococcus* spp., resistance to CLI, LNZ, and CIP was low, whereas resistance to TET, PEN, and GEN exceeded 50%. In another study, higher resistance rates to TET and PEN have been reported [94,121], likely reflecting their frequent use to fight infections by *Streptococcus* [122,123]. Nevertheless, TET resistance observed here was lower than studies reporting > 90% resistance [94,113,124] (Figure 4).

The literature reports a broad antibiotic-resistance development related to synergistic use of antibiotics to treat infections, such as the frequent use of penicillin with gentamicin to manage severe *Streptococcus* infections [125,126,127,128,129].

**Figure 5:**
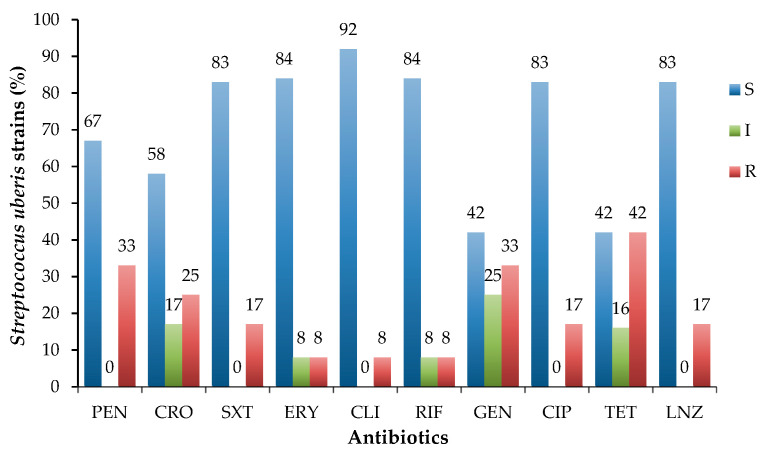
Antibiotic susceptibility profile of *Streptococcus uberis* strains. PEN: penicillin; CRO: ceftriaxone; SXT: sulfamethoxazole; ERY: erythromycin; CLI: clindamycin; RIF: rifampicin; GEN: gentamicin; CIP: ciprofloxacin; TET: tetracycline; LNZ: linezolid; S: susceptible; I: intermediate; R: resistant. n = 12.

In *Streptococcus uberis*, resistance was notable for PEN (67%), TET (42%), and GEN (42%) (Figure 5), which is higher than that reported in the USA, Switzerland, Korea, and Estonia, with reported resistance levels of 0.4–8.1% to PEN [71,128,129,130,131,132,133,134,135]. These could be in accordance with the periodic increase in resistance reported since 1994 by the common use of PEN for treating and preventing dairy cow infectious diseases [71,136,137,138,139,140,141].

For TET, the resistance levels were comparable with Brazil and Portugal, with 50% and 42% resistance to TET in *S. uberis*, respectively, in bovine milk samples [123,142]. On the other hand, an 82.02% TET resistance was reported in Thailand [143]. Although TET is not always a first-line option for SBM or BM, prolonged exposure and slow degradation in the body can promote the emergence or amplification of resistant bacteria [144,145].

Overall, the susceptibility profile variation between studies can depend on the antibiotics used to manage SBM in different regions and on the application of breakpoints derived from humans [142]. Therefore, establishing periodic and local SBM surveillance is critical to define the antibiotic resistance landscape and guide effective control measures [142].

### 3.4. Antibiotic Resistance and Virulence Genes Detection

In *Staphylococcus*, *mecA* was detected in 7.8% of isolates and significantly associated with OXA/FOX phenotypic resistance (*p*-value = 0.01) (Table 5). Here, the *mecA* presence was lower than the 18.8% reported in Bangladesh [31,140]. Furthermore, several FOX/OXA-resistant isolates lacked the *mecA* gene, whereas one *mecA*-positive isolate was phenotypically susceptible. Such discordance between the phenotype and genotype could reflect allelic variants associated with methicillin resistance, such as *mecC*, or the involvement of alternative resistance determinants [146].

The *nuc* gene was found in 29.9% of isolates, mostly grouped in *S. aureus* (Table 5). This was expected because *nuc* encodes a thermonuclease that is produced by nearly all *S. aureus* strains [147]. The *nuc* thermonuclease virulence factor is used as a diagnostic tool for *S. aureus* identification in SBM, as well as clinical samples [30,148,149,150].

The genetic determinants of the virulence factor Panton–Valentine leukocidin (PVL), i.e., *lukSF PV* genes, were identified in 6.5% of strains (Table 5). A study has reported 9% of isolates presenting the PVL genes in intramammary infections of Ethiopian dairy cows [47]. In Germany, all MRSA isolates from SBM were negative for PVL [151], and 1–10% of methicillin-susceptible *S. aureus* isolates presented these genes [152]. By contrast, higher frequencies have been described in countries such as Rwanda, with 12.4–21.4% of SBM-associated *Staphylococcus* strains showing PVL-positive genes [153]. The occurrence of PVL virulence genes in bovine samples has been attributed to anthroponotic transmission since MRSA infections are strongly linked to healthcare settings [154,155,156,157]. Moreover, healthy human carriers can act as sources of transmission to cattle, enabling the spread from operators to cows and subsequent SBM development [158,159,160,161].

The *tetL* resistance gene was detected in 3.9% of *Staphylococcus* isolates (Table 5). This gene did not show a significant association with phenotypic TET resistance (*p*-value = 0.069). In addition, TET-resistant isolates lacking *tetL* were identified, as well as *tetL*-positive isolates susceptible to TET. In Hatay (Turkey), phenotypic resistance to TET was observed in 41% of *S. aureus* clinical isolates, where 42.4% of these carried *tetK* and *tetM* genes [162]. Furthermore, in Colombia, a significant association between *tetK* or *tetM* and phenotypic TET resistance in milk isolates was identified [145]. The detection of phenotypically TET-resistant isolates without *tetL* could reflect the contribution of alternative tetracycline resistance determinants [163,164].

ERY resistance is commonly attributed to the macrolide resistance genes *ermB*, *ermC*, and *msr* [165]. In this work, *ermB* was found in 6.5% of *Staphylococcus* isolates (Table 5) and showed a positive association with phenotypic ERY resistance (*p*-value = 0.020) (Figure 3). Consistently, a report in Mexico identified 6.5% of *S. aureus* as being ERY resistant, correlating with the presence of *ermB* and *ermC* genes [30]. Moreover, isolates with intermediate susceptibility to ERY carrying the *ermB* gene were found here. This phenotype has been linked to an altered *23S rRNA* gene structure via methylation, yet this was not studied here [166,167]. However, *Staphylococcus* ERY-resistant isolates with no amplification of *ermB* were also identified here. This could reflect the presence of alternative macrolide resistance gene variants, such as *ermA* or *ermC*, which were not analyzed here [168].

For *Streptococcus*, 18% of isolates carried the *ermB* gene (Table 5), in accordance with the phenotypic results of ERY phenotypic resistance (*p*-value = 0.024) (Figure 4). *ermB*-positive isolates were identified in *S. agalactiae* and *S. uberis*. Notably, this level is substantially lower than those reported elsewhere (58.8–83.33%) [123,142,169].

Finally, the *blaZ* gene, responsible for penicillin resistance in *Streptococcus*, was detected in 50% of isolates in three species (*S. uberis, S. dysgalactiae*, and *S. agalactiae*) (Table 5). There was no statistically significant association between the presence of *blaZ* gene and phenotypic penicillin resistance (*p*-value = 1), despite the 50% prevalence of penicillin resistance observed here (Figure 4). Among the eleven *blaZ*-positive isolates, five were resistant to penicillin, whereas six were susceptible, perhaps due to the lack of *blaZ* gene expression [Table 6]. High penicillin resistance levels in cattle are well documented worldwide and may reflect the extensive use of β-lactams for mastitis therapy and prophylaxis [135]. For example, some studies have reported the presence of *blaZ* in 90.7% of isolates [165]. Despite β-lactams playing an important role in SBM control, inappropriate use promotes the selection and spread of resistance [170]. Moreover, while β-lactam and β-lactamase inhibitors are effective in human medicine, their application in the dairy industry is costly, limiting the practical adoption and leading to low economic profitability for milk production [33,171].

However, although this study successfully characterized the predominant *Staphylococcus* and *Streptococcus* genera and their resistance profiles, the cross-sectional design and sampling within a defined timeframe limit the ability to capture the temporal dynamics of antimicrobial resistance development. Therefore, future studies are needed to better understand these dynamics. Expanding the sample size and incorporating advanced genomic approaches (e.g., whole-genome sequencing) would also strengthen surveillance by enabling higher-resolution tracking of strain dissemination and resistance determinants.

## 4. Conclusions

This study provides a local baseline of SBM etiology and AMR in northern Ecuador, showing that *S. aureus* and *S. uberis* are leading species in the herds investigated. The combined use of cultures, MALDI-TOF, and PCR offers a robust overview of circulating pathogens and their key resistance determinants in the study area. *Staphylococcus* exhibited high resistance to FOS, while *Streptococcus* showed elevated resistance to PEN and TET, which are patterns that represent the historical and ongoing antimicrobial use in these settings. The detection of *mecA* in *Staphylococcus* and *blaZ* and *ermB* in *Streptococcus* is significantly associated with the corresponding resistance phenotypes, although the incomplete concordance, particularly for tetracycline, highlights the need to screen additional resistance determinants in future surveys. Furthermore, these findings support the need for culture- and susceptibility-guided therapy over empirical treatment and reinforce the importance of antimicrobial stewardship. Routine, local AMR surveillance integrating phenotypic testing with molecular detection should be implemented; stronger milking hygiene, such as consistent post-milking teat disinfection and single-use drying materials, is also needed to reduce the infection pressure, antibiotic use, and One Health risk.

## Figures and Tables

**Table 1:** CMT score interpretation.

Thickening/Gel Formation	Score	Interpretation
None	0	Negative
Mild	T	Traces (minor infection risk)
Moderate	+	Positive-low (subclinical mastitis)
High	++	Positive-medium (subclinical mastitis)
Solidified	+++	Positive-high (subclinical mastitis near the clinical expression)

**Table 2:** Zone diameter breakpoints used for the interpretation of antibiotics tested in *Staphylococcus* spp. and *Streptococcus* spp.

Microorganism	Antimicrobial Agent	S (mm)	I (mm)	R (mm)	Reference
*Staphylococcus* spp.	Cefoxitin	≥22	—	≤21	[25]
	Oxacillin	≥13	11–12	≤10	[25]
	Sulfamethoxazole	≥16	11–15	≤10	[25]
	Erythromycin	≥23	14–22	≤13	[25]
	Clindamycin	≥21	15–20	≤14	[25]
	Rifampicin	≥20	17–19	≤16	[25]
	Gentamicin	≥15	13–14	≤12	[25]
	Ciprofloxacin	≥21	16–20	≤15	[25]
	Tetracycline	≥19	15–18	≤14	[25]
	Fosfomycin	≥22	—	≤21	[26]
	Linezolid	≥26	23–25	≤22	[25]
*Streptococcus * spp.	Penicillin	≥24	—	≤23	[25]
	Ceftriaxone	≥27	25–26	≤24	[25]
	Sulfamethoxazole	≥16	—	≤15	[26]
	Erythromycin	≥21	16–20	≤15	[25]
	Clindamycin	≥19	16–18	≤15	[25]
	Rifampicin	≥24	19–23	≤18	[26]
	Gentamicin	≥16	13–15	≤12	[25]
	Ciprofloxacin	≥21	16–20	≤15	[25]
	Tetracycline	≥23	19–22	≤22	[25]
	Linezolid	≥19	—	≤18	[25]

S: susceptible; I: intermediate; R: resistant.

**Table 3:** Antibiotic resistance and virulence genes.

Microorganism	Gene		Primers (5′–3′)		Annealing Temperature (°C)	Reference
	Name	Function	Forward	Reverse		
*Staphylococcus* spp.	*mecA*	Methicillin resistance	GTAGAAATGACTGAACGTCCGATAA	CCAATTCCACATTGTTTCGGTCTAA	55	[28,29]
	*nuc*	Endonuclease activity	GACTATTATTGGTTGATCCACCTG	GCCTTGACGAACTAAAGCTTCG	54	[30]
	*lukSF-PV*	Panton–Valentine leukocidin (PVL)	ATCATTAGGTAAAATGTCTGGACATGATCCA	GCATCAAGTGTATTGGATAGCAAAAGC	55	[31]
*Staphylococcus* spp. and *Streptococcus* spp.	*tetL*	Tetracycline resistance	TGAACGTCTCATTACCTG	ACGAAAGCCCACCTAAAA	52	[32]
	*ermB*	Macrolides resistance	GAAAAGGTACTCAACCAAATA	AGTAACGGTACTTAAATTGTTTAC	54	[32]
*Streptococcus* spp.	*blaZ*	Penicillin resistance	AAGAGATTTGCCTATGCTTC	GCTTGACCACTTTTATCAGC	55	[33]

**Table 4:** Prevalence of subclinical mastitis in cattle.

Dairy Farm	Milking Method	CMT Outcome (#)		Mastitis Prevalence (%)
		−	+	
1	Mechanical	9	9	50
2	Manual	5	6	54.5
3	Mechanical	14	16	53.3
4	Mechanical	31	6	16.2
5	Mechanical	54	22	28.9
6	Mechanical	59	21	26.3
Total		172	80	31.7

**Table 5:** Isolates with antibiotic resistance and virulence molecular determinants identified using PCR.

Microorganism			Isolates with Antibiotic Resistance and Virulence Genes (n)					
Genus (n)	Species (n)		*nuc*	*lukSF-PV*	*mecA*	*tetL*	*ermB*	*blaZ*
*Staphylococcus* (77)	*S. aureus*	(28)	21	3	4	1	0	ND
	*S. warneri*	(16)	1	0	1	0	1	ND
	*S. epidermidis*	(12)	0	1	0	1	2	ND
	*S. hominis*	(10)	1	0	1	0	1	ND
	*S. haemolitycus*	(4)	0	0	0	1	0	ND
	*S. auricularis*	(2)	0	1	0	0	0	ND
	*S. hyicus*	(2)	0	0	0	0	0	ND
	*S. caprae*	(1)	0	0	0	0	0	ND
	*S. equarum*	(1)	0	0	0	0	1	ND
	*S. simulans*	(1)	0	0	0	0	0	ND
			29.9%	6.5%	7.8%	3.9%	6.5%	
*Streptococcus* (22)	*S. uberis*	(12)	ND	ND	ND	0	2	8
	*S. agalactiae*	(4)	ND	ND	ND	0	2	2
	*S. dysgalactiae*	(4)	ND	ND	ND	0	0	1
	*Streptococcus* sp.	(2)	ND	ND	ND	0	0	0
						0%	18.2%	50%

ND: no data.

**Table 6:** Inhibition zone diameters observed for penicillin in the *Streptococcus* spp. strains.

Zone Diameters	32	21 *	15 *	15 *	6 *	18 *	32	30	23 *	23 *	40	46	42	16 *	23 *	36	38	31	38	43	23 *	17 *
*blaZ* gene presence	−	+	+	−	−	+	−	−	−	−	+	+	+	+	+	−	+	−	+	+	−	−

+: positive; −: negative; *: resistant strain.

## Data Availability

The original contributions presented in this study are included in the article. Further inquiries can be directed to the corresponding author.
